# CMOS integration of inkjet-printed graphene for humidity sensing

**DOI:** 10.1038/srep17374

**Published:** 2015-11-30

**Authors:** S. Santra, G. Hu, R. C. T. Howe, A. De Luca, S. Z. Ali, F. Udrea, J. W. Gardner, S. K. Ray, P. K. Guha, T. Hasan

**Affiliations:** 1 Department of Physics, Indian Institute of Technology, Kharagpur, 721302, India; 2 Cambridge Graphene Centre, University of Cambridge, Cambridge, CB3 0FA, UK; 3 Department of Engineering, University of Cambridge, Cambridge, CB3 0FA, UK; 4 Cambridge CMOS Sensors Ltd., Cambridge, CB4 0DL, UK; 5 School of Engineering, University of Warwick, Coventry, CV4 7AL, UK; 6 E & ECE Department, Indian Institute of Technology, Kharagpur, 721302, India

## Abstract

We report on the integration of inkjet-printed graphene with a CMOS micro-electro-mechanical-system (MEMS) microhotplate for humidity sensing. The graphene ink is produced via ultrasonic assisted liquid phase exfoliation in isopropyl alcohol (IPA) using polyvinyl pyrrolidone (PVP) polymer as the stabilizer. We formulate inks with different graphene concentrations, which are then deposited through inkjet printing over predefined interdigitated gold electrodes on a CMOS microhotplate. The graphene flakes form a percolating network to render the resultant graphene-PVP thin film conductive, which varies in presence of humidity due to swelling of the hygroscopic PVP host. When the sensors are exposed to relative humidity ranging from 10–80%, we observe significant changes in resistance with increasing sensitivity from the amount of graphene in the inks. Our sensors show excellent repeatability and stability, over a period of several weeks. The location specific deposition of functional graphene ink onto a low cost CMOS platform has the potential for high volume, economic manufacturing and application as a new generation of miniature, low power humidity sensors for the internet of things.

Humidity sensors are employed today in a wide range of applications, including in environmental monitoring, automotive, industrial process, healthcare, agriculture, and increasing indoor air quality in smart buildings. Over the years, a variety of transduction techniques has been reported for humidity sensors, including the use of surface acoustic wave (SAW)^1^,^2^,^3^, resistive^4^,^5^, capacitive^6^, optical fibre^7^, field effect transistor^8^,^9^, and quartz crystal microbalance^10^. Sensors based on some of these transduction techniques are available on the market, such as the SHTC1 digital humidity sensor^11^. Among these approaches, capacitive technique is the most widely used for humidity sensing because of its linear response to humidity over a wide range^12^. Capacitive sensing makes use of a thin dielectric layer, typically a polymer, to adsorb/desorb water molecules and thus detect changes in the capacitance (*i.e*. electrical permittivity) for humidity sensing^12^. However, hysteresis is a major drawback in polymer-based capacitive humidity sensors. This is due to the cluster of water adsorbed inside bulk polymers that may cause deformation and instability of the sensing polymer layer, eventually reducing the lifetime of the sensor^12^. In addition, most of the current generation of chemical, gas and humidity sensors uses semi-automated manufacturing, increasing the overall production cost^13^. On the other hand, resistive humidity sensors are easier to read out with basic circuitry compared to other types of sensors. Indeed, resistive sensors with integrated CMOS on a single silicon chip can reduce the production cost significantly. In recent years, various materials have been studied as sensing materials for resistive humidity sensors such as polymers^1^,^14^, metal oxides^8^, carbon nanotubes^15^,^16^ and graphene oxide-based polymer/polyelectrolyte nanocomposites^5^,^17^. Among these, nanomaterials are particularly attractive because of their high surface area to volume ratio, promising high sensitivity and fast response times.

Sensors available on the market are typically bulky or usually have high power consumption^18^. CMOS is suitable as an underlying platform for the development of economic, compact and low power sensing devices. It is a mature, reliable technology and most importantly, it makes integration of sensors with electronics possible. However, to take advantage of CMOS miniaturization and single chip solution, their small active sensing areas need to exploit high sensitivity materials. Nanomaterials are therefore an ideal candidate for a low cost CMOS sensing platform.

With its 2-dimensional (2d) structure, high specific surface area and in particular, sensitivity of its electrical properties to environmental perturbations, graphene has attracted significant attention in various sensing applications including humidity^5^ and gas sensing^19^,^20^,^21^,^22^,^23^. Graphene can be prepared via various methods, the most widely exploited ones being mechanical cleavage^24^ and chemical^25^ and shear force assisted exfoliation of graphite^26^ and chemical vapour deposition (CVD)^27^. Among these, micromechanical cleavage^24^ produces the highest quality flakes, although at extremely low yield^28^. It is thus only widely used in fundamental studies of graphene and related 2d materials^28^. Though CVD has, in recent years, been scaled up to produce large area, high quality graphene^29^, the high temperature growth process is not ideal for device fabrication and integration. In addition, CVD grown mono- or few-layer graphene has limited exposed surface area and edges (*i.e*. active area) compared to graphene produced by solution processing, a key requirement for sensing applications. Solution based strategies, such as ultrasonic assisted liquid phase exfoliation (UALPE) produces high quality, defect free, pristine graphene nanoflakes at room temperature^30^. The lateral size of the graphene nanoflakes is typically several hundred nanometres, offering high surface area and edges, making them ideal active materials for sensors. Thus far, the demonstrations of sensors based on graphene or graphene oxide (GO) are mostly based on non-CMOS platform such as on glass/ZnO^1^, glass^6^, polyamide substrate^17^, ceramic substrate^31^ and polyethylene naphthalate^32^.

Here, we demonstrate resistive CMOS MEMS devices, fully integrated with an inkjet-printed chemically pristine graphene-polyvinyl pyrrolidone (PVP) composite sensing layer for humidity sensing. Our graphene ink formulation allows selective area deposition and room temperature curing after printing. The conductive graphene flakes, interspersed inside the insulating, hygroscopic PVP form a 3-dimensional (3d) percolating network that responds to adsorbed moisture by the polymer with a change in electrical resistivity, allowing humidity sensing.

## Results

### Microhotplate design and fabrication

**Figure 1:**
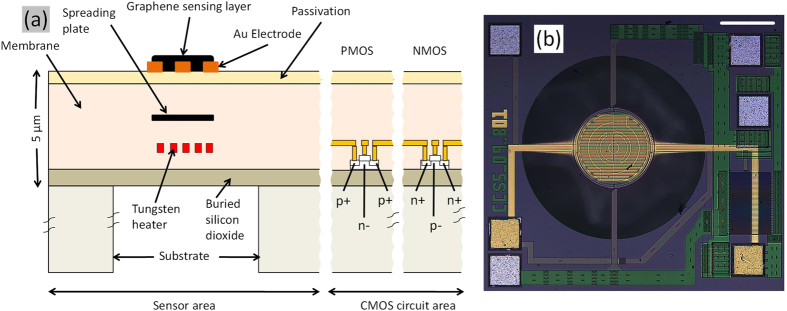
(**a**) Cross sectional view (not to scale) and (**b**) Optical micrograph of the CMOS device (the scale bar at top right is 200 *μ*m).

A key component of our resistive humidity sensor is the CMOS MEMS based microhotplate (*μ*HP) structure. Details of the *μ*HP structure are reported elsewhere^33^,^34^. A cross section view of the sensor structure is shown in Fig. 1(a). An optical microscope image of the fabricated device is shown in Fig. 1(b). The silicon die measures 1 mm × 1 mm. It is designed using a 1.0 *μ*m Silicon on Insulator (SOI) CMOS process technology and fabricated in a commercial foundry, followed by deep reactive ion etching (DRIE) to release the thin membrane. The processing employs SOI wafers with 1 *μ*m buried oxide, 0.25 *μ*m SOI layer and 3 metallization layers. The *μ*HP structure typically consists of an embedded, 0.3 *μ*m thick, resistive tungsten microheater (metal layer 1), 0.3 *μ*m thick heat spreader plate (metal layer 2), and a top gold layer for interdigitated sensing electrodes (IDEs). The diameters of the circular heater and membrane structures are 250 and 600 *μ*m, respectively. The heater is fabricated during the CMOS process while the top gold electrodes and corresponding tracks are deposited as a post CMOS process in the same commercial foundry. The tungsten heater controls the operating temperature of the membrane and graphene-based sensing layer. The IDEs underneath the sensing layer are used to measure the change in resistance due to humidity exposure. Use of tungsten in the heater allows the device to operate at a very high temperature (up to 750 °C), if required, for example, when oxide-based sensing materials are used. Gold is used as the electrode material because of its chemical inertness (and hence, unchanged conductivity over prolonged use under various temperature and humidity conditions) compared to commonly used aluminum in this SOI process. The silicon underneath the *μ*HP is etched away, using the dioxide layer as the etch stopper, at a wafer level by DRIE technique. This forms a 4.5 *μ*m silicon dioxide: SiO_2_ (4 *μ*m)/silicon nitride: Si_3_N_4_ (0.5 *μ*m) membrane structure onto which the microheater and electrodes are suspended. The membrane structure reduces DC power consumption of the sensing device to <5 mW when used for humidity sensing in this work. The heating temperature is uniformly confined over the microheater region, due to the buried heat spreader^33^. The temperature decreases rapidly away from the heater region and is at close to room temperature at the membrane rim, allowing reliable temperature independent on-chip circuit performance^35^.

**Figure 2:**
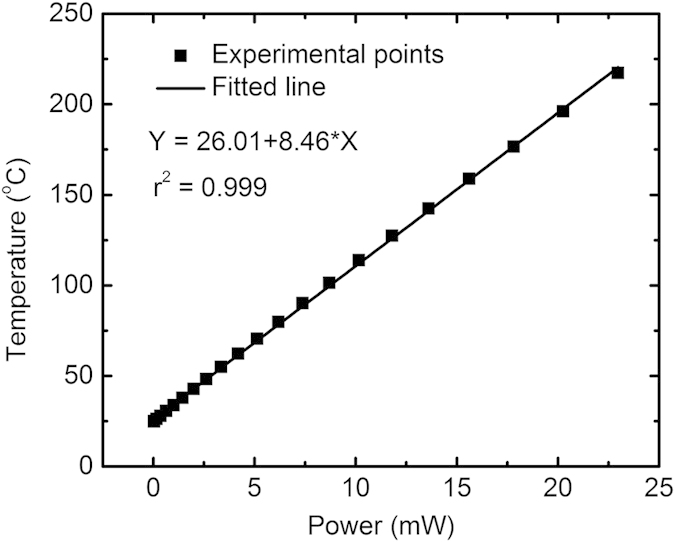
Temperature versus input power testing with the correlation coefficient *r* given.

The characteristic power versus temperature plot (up to 217 °C) of the *μ*HP device is given in Fig. 2. To calculate the power consumption, the resistive *μ*HPs are first calibrated to up to 300 °C using a computer controlled high temperature chuck (Signatone S-1060R-6TG). Two temperature coefficients of resistance (*α*, *β*) are calculated from the measured value using the relationship: 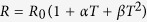, where 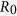 is the resistance of the heater at room temperature 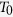, and 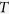 is the temperature increase. The values of *α* (2.05 × 10^−3^ *K*^−1^) and *β* (0.2 × 10^−6^ *K*^−2^) are very closely matched to the values provided by the CMOS foundry. A constant current is supplied to the 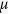
HP and the voltage is measured across the heater. From the constant current and measured voltage, the power supplied to the devices is calculated. The corresponding heater temperature is calculated from the change in resistance using 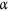, 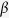 values estimated during the calibration. Over small temperature changes, a linear function is fitted through the experimental points of power versus temperature plot, yielding an electrothermal transduction efficiency of 8.46 °C/mW; Fig. 2.

### Formulation of graphene-polymer ink

The first step towards ink formulation is exfoliation of graphite into graphene flakes. UALPE starts with mixing bulk graphite crystals into a solvent. The ultrasound causes high-frequency pressure variations and formation of microcavities in the solvent. Collapse of these microcavities produces high shear forces, exfoliating mono-, bi- and few-layer flakes from the bulk crystals by overcoming the interlayer van der Waals forces^36^. Since the UALPE process does not involve chemical pre- or post-treatment, the dispersed graphene flakes are chemically pristine. Note that, when experimental parameters such as sonication vessel and solvent volume are kept fixed^37^, solvent viscosity plays an important role in cavitation (higher viscosity requires higher acoustic pressure to create cavitation^38^) and hence, the UALPE process. However, in reality, a large majority of common solvents have a viscosity ranging from 1 to 5 mPa.s at room temperature, which have no strong effect on the exfoliation. Instead, the dispersability and stability of graphene in the solvents are largely governed by their intermolecular interactions. This has been explained through matching the degree of the Hansen solubility parameters 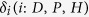 of graphene and solvents, where 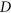, 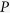 and 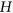 are the dispersive, polar and hydrogen-bonding components, respectively^39^. It was experimentally observed that effective solvents for the exfoliation and stabilization of graphene should possess Hansen solubility parameters close to 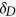 = 18 MPa^1/2^, 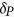 = 9.3 MPa^1/2^ and 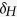 = 7.7 MPa^1/2^. N-methyl-2-pyrrolidone (NMP) is an example of such effective solvents^30^ (with 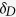 = 18 MPa^1/2^, 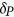 = 12.3 MPa^1/2^ and 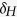 = 7.2 MPa^1/2 ^)^40^. However, the surface tension of the solvents (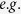 NMP has a surface tension of ~40.7 mNm^−1^) suitable for graphene typically poses a challenge for inkjet printing on low energy surfaces^26^ such as the 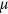
HP membrane (the surface energy of Si_3_N_4_ is estimated as ~40 mNm^−1^)^41^. This results in poor wetting of the substrates^42^ and inconsistent coating^43^, leading to non-uniform deposition of graphene and ‘coffee-ring effect’ after solvent evaporation.

It is thus required to develop graphene inks with low surface tension solvents to allow for good wetting. Alcohols, in particular, are attractive for this purpose. In addition, their low boiling point allows rapid drying of ink after deposition, ensuring uniform coating of graphene flakes. Indeed, they are commonly used as the primary or secondary solvent in the majority of graphics and functional inks^44^. Among the common alcohols, isopropyl alcohol (IPA) can exfoliate graphene, but with meta-stable dispersion^45^. This is primarily due to its mismatched Hansen solubility parameters (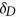 = 15.8 MPa^1/2^, 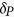 = 6.1 MPa^1/2^ and 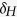 = 15.4 MPa^1/2^) with graphene. Therefore, pure IPA based graphene dispersion cannot be used for inkjet printing. UALPE with ionic surfactants and nonionic polymers are commonly used strategies to stabilize graphene^46^,^47^. This is typically achieved through steric hindrance and favourable enthalpic interactions with graphitic surface^48^. Here, we use PVP as the stabilizer. Indeed, PVP is a polymer analog to NMP with the N-substituted pyrrolidone rings similar to that of NMP^49^,^50^, and has been used before to stabilize carbon nanostructures, such as nanotubes in different solvents^49^,^51^,^52^. In addition, PVP is highly hygroscopic, swelling when absorbing moisture in humid environments^53^. PVP is also an electrically insulating polymer but can be made conductive by addition of graphene through percolation^54^. The hygroscopic nature and moisture-induced swelling with conductivity change in graphene-PVP composites make our formulated graphene-PVP-IPA ink (henceforth, we use the term ‘graphene ink’) very attractive for humidity sensing.

We ultrasonicate 100 mg of graphite (Sigma-Aldrich, 100 mesh flakes) with 1.5 mg PVP (Sigma-Aldrich, average molecular weight 10,000 Da) in 10 mL IPA for 12 hours at ~15 °C. This starting PVP concentration enables stabilization as well as desired ink rheological properties specific to our inkjet printer nozzle discussed below. The resultant dispersion is centrifuged for 1 hour at 4,030 rpm (~1540 *g*), sedimenting the unexfoliated graphite. The upper 70% of the dispersion, enriched with mono- and few-layer graphene nanoflakes, is decanted for analysis and experiment.

**Figure 3:**
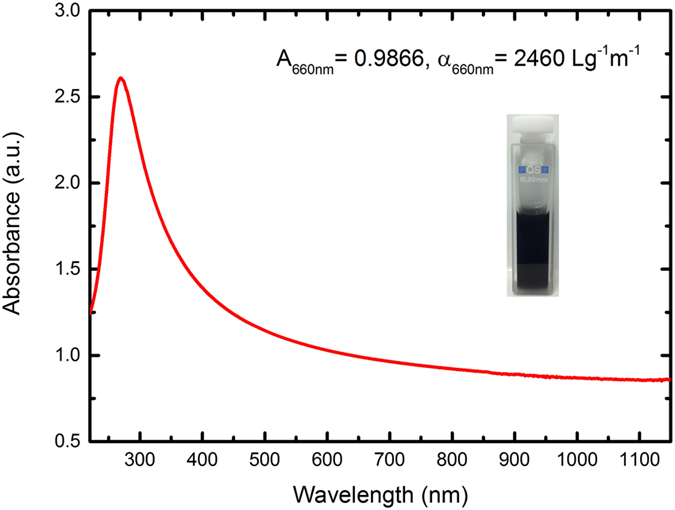
Optical absorption spectrum of graphene ink. To avoid scattering loss, the dispersion is diluted to 10 vol% for UV-Vis-NIR measurement. The inset is the cuvette containing original graphene dispersion.

Optical absorption spectroscopy is used to estimate the concentration of the dispersed graphene using the Beer-Lambert law (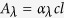), where 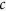 is the graphene concentration (gL^−1^) and 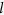 is the distance the light passes through the dispersion (m). 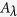 and 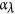 are the absorption (a.u.) and material dependent optical absorption coefficient (Lg^−1^m^−1^) at wavelength 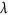 (nm), respectively. Figure 3 shows the optical absorption spectrum of the dispersion diluted to 10 vol% to avoid scattering losses during absorption measurement^55^. The inset of Fig. 3 shows a photograph of a cuvette containing the undiluted graphene dispersion. The spectrum is mostly featureless as expected, due to the linear dispersion of Dirac electrons^56^,^57^. The peak in the UV region is a signature of the van Hove singularity in the graphene density of states^58^. Using *α*_660 *nm*_ = 2460 Lg^−1^m^−1 ^47, we estimate the concentration of graphene in the undiluted dispersion as 0.40 gL^−1^. We note that the graphene ink is stable, without forming any visible aggregation over several months.

**Figure 4:**
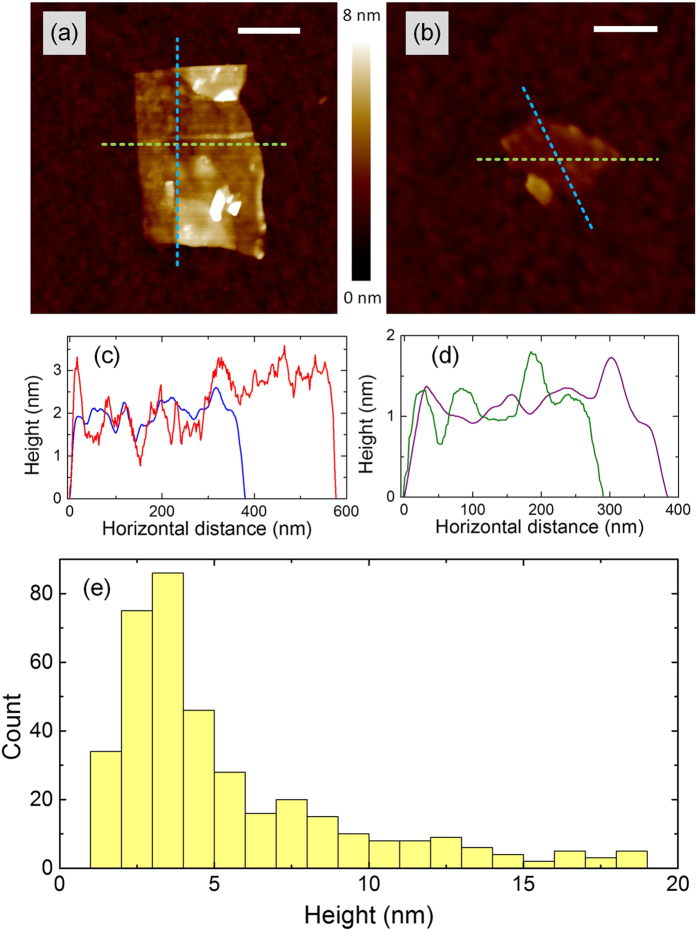
AFM characterizations of graphene flakes: (**a**) and (**b**) micrographs of typical flakes, the scale bar is 200 nm; (**c**) and (**d**) height variations across (**a**) and (**b**), respectively; (**e**) thickness distribution.

The graphene flakes are characterized with Atomic Force Microscopy (AFM). The sample for AFM is prepared by dip-coating a Si/SiO_2_ wafer into a graphene dispersion diluted to 2 vol% by pure IPA. Though the concentration of PVP in this diluted dispersion is ~3.8 × 10^−4^ wt%, the residual polymer prevents accurate measurement of the flake dimensions. The sample is therefore annealed at 400 °C for 30 min. This temperature is chosen because PVP starts to decompose at this temperature in air^59^ while the exfoliated graphene flakes remain stable^60^,^61^. The annealed sample is imaged with a Bruker Dimension Icon AFM in ScanAsyst^TM^ mode, using a silicon cantilever with a Si_3_N_4_ tip. Micrographs of typical flakes are shown in Fig. 4(a,b), along with the height variations across the samples; Fig. 4(c,d). The thickness distribution of flakes is also measured; Fig. 4(e). This shows that 63% of the flakes have thicknesses <5 nm, corresponding to <13 layers, assuming ~0.7 nm measured thickness for the bottom layer and ~0.35 nm increase in thickness for each additional layer^62^. The average flake lateral dimension is ~204 nm. Note that the number of layers obtained in IPA/PVP is higher than what is typically obtained by UALPE of graphite in water/SDC, with ~26% mono- and ~22% bi-layer, and with 300–600 nm average lateral dimension from similar experimental parameters^47^.

### Inkjet printing of graphene

We next consider deposition of our graphene ink to form a graphene-PVP thin film composite as the active sensing layer over the IDEs of the 250 *μ*m diameter CMOS 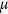
HP. For this, large area techniques such as spray-, spin- or dip-coating are unsuitable, as they do not offer selective area deposition directly on to the IDEs. We use drop-on-demand inkjet printing technique for the deposition of active materials on to the IDEs. It is a digital printing technique, where single ink droplets are ejected from an ink chamber in response to individual electrical impulses^26^. Inkjet printing also offers selective area deposition across the X-Y plane of a substrate. The inkjet printer used in this work is a DMP-2831 Dimatix Printer. The printing nozzle has a diameter of 22 *μ*m. The volume of individual droplets from this nozzle is ~10 pL. We achieve printing resolutions of ~100 *μ*m with this set-up onto the IDEs without any surface modification. By controlling the number of droplets and the area of deposition, inkjet printing thus enables us to deposit a well defined volume of ink for humidity sensing only onto the IDEs.

**Figure 5:**
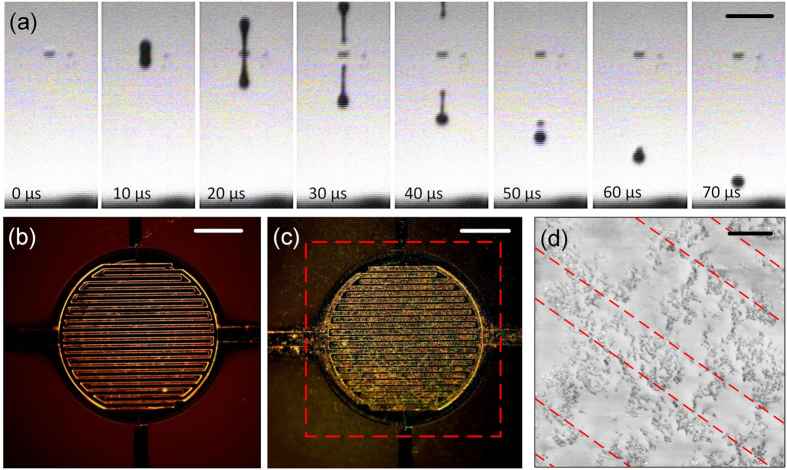
(**a**) Stable jetting sequence of graphene ink, the scale bar is 100 *μ*m; (**b**) Dark field optical microscope image of the IDEs on CMOS *μ*HP (**b**) without graphene (**c**) with graphene-PVP deposited on to IDEs, with the targeted printing area marked by dashed lines, the scale bar is 100 *μ*m; (**d**) SEM image of a small area on the CMOS *μ*HP with graphene-PVP deposited; the IDEs are marked by dashed lines and the scale bar is 3 *μ*m.

A stable drop generation (single droplet generation for each electrical impulse, without the formation of satellite droplets) and jetting of ink (avoiding deviation of droplet trajectory) is of primary importance for high quality inkjet printing. Otherwise, unstable jetting may lead to uncontrolled amount of ink deposition on to undesired locations. In inkjet printing, a figure of merit, 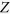, is commonly used to consider the printability of inks and is defined as: 
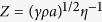
, where 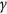 is surface tension of the ink (mNm^−1^), 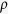 is the density of ink (gcm^−3^), 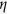 is the viscosity of the ink (mPa.s) and 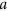 is the nozzle diameter (*μ*m). As a rule of thumb, it is commonly accepted that 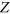 value should be <14 for the case of drop-on-demand inkjet printing to avoid secondary or satellite droplets^26^,^63^. Meanwhile, it should be >1 to optimize droplet formation or ejection to avoid long-lived filament formation, which may result in poor positional accuracy and printing resolution^26^,^63^,^64^. The value of 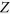 typically falls within 1–10 for commercial inkjet printable inks^65^. We stress that the acceptable range of 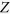 values should be established by experiments and numerical simulations and should be considered as a guide only. Indeed, inks with values outside this range may also be printable, in particular, by controlling the shape and intensity of the electrical pulses for droplet generation. To determine the *Z* value of our graphene ink, we use pendant drop measurement and parallel plate rheometry to measure 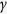 and 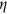, respectively at room temperature. We measure 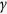 ~ 28.0 mNm^−1^ and 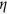 ~ 2.34 mPa.s. The density is measured as 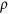 ~ 0.8 gcm^−3^. With *a* = 22 *μ*m, we calculate *Z* ~ 9.48, falling into the recommended range for stable jetting. This indicates the formulated ink is suitable for inkjet printing. A stable jetting without the formation of satellite droplet or long filament is thus expected. This is experimentally confirmed by high speed jetting sequence images presented in Fig. 5(a).

The drying process of the deposited ink is critical to ensure uniform coverage after the carrier solvent is evaporated. After impacting the substrate, the wettability of the ink droplets carrying stably dispersed nanoparticles (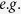 graphene) defines consistency of the particle coating on to the substrate^43^. During the drying process of a droplet, a finite contact edge is formed at the edge of the solid-solvent interface. This enhances the transport of the solvent, promoting faster evaporation at the edges than in the central area of the droplet. Meanwhile, the difference in evaporation speeds results in a convective flow within the droplet from the center towards the edges (Marangoni effect)^66^. This transports the dispersed materials to be deposited at these edges. These deposited dried materials prevent the contact edge from receding, ‘pinning’ it at its original position. This further promotes the deposition of particles as the solvent evaporates, leading to a non-uniform coating at the edges, commonly termed as the ‘coffee ring effect’^67^,^68^. The low surface tension of our ink ensures a good wetting, and the low boiling point of IPA (82.6 °C) guarantees a quick evaporation at room temperature, leading to a consistent and uniform coverage of graphene flakes across the printed area. Figure 5(c) shows the dark field optical microscope images of the CMOS device with graphene deposited. The bright spots in Fig. 5(c) indicate graphene flakes in the PVP matrix, demonstrating an even deposition of graphene across the IDEs. We also investigate the structure of the deposited graphene-PVP using scanning electron microscopy (SEM); showing evenly interspersed graphene flakes (darker spots) on the CMOS 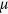
HP; see Fig. 5(d).

### Humidity sensing

**Figure 6:**
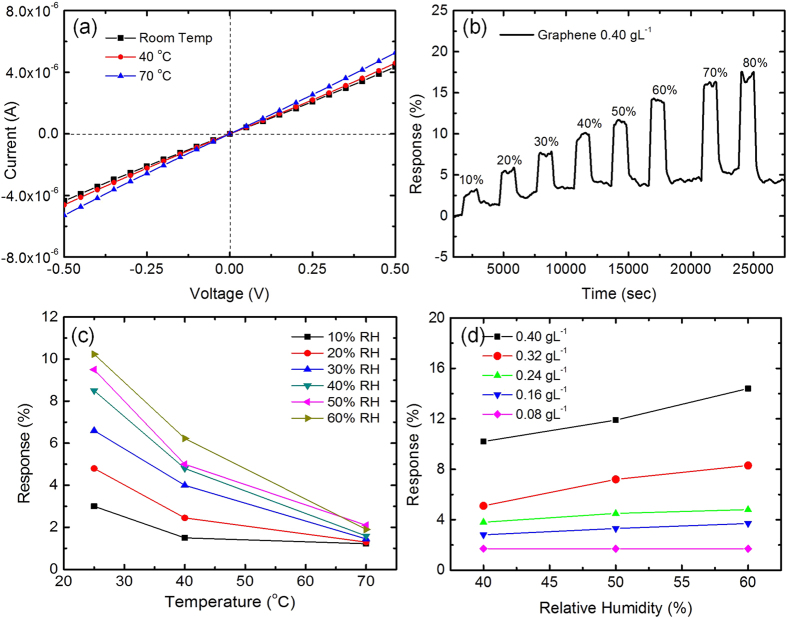
(**a**) Current versus voltage of humidity sensor at three different temperatures; (**b**)Humidity sensing response at room temperature with eight different 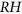 levels; (**c**) Sensing response under three different temperature conditions (room temperature, 40 °C and 70 °C); (**d**) Humidity sensing varies with the amount of deposited graphene.

Evaporation of IPA forms a graphene-PVP composite sensing layer on to the IDEs. Within the graphene-PVP composite, the graphene flakes are randomly distributed. The electrical behaviour of randomly arranged conducting objects in an insulating thin film matrix can be described by percolation theory in 3d 69. When continuous pathways of the conducting objects are not formed, the conductivity of the matrix is zero. As the conductive pathways start building up, electrical conductivity becomes non-zero and increases with an increasing number of conducting pathways following the relation:


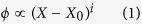


where *ϕ* is the conductivity, 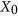 is the critical density of the conducting objects above which the density of the objects 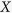 results in conductivity and 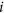 is the percolation coefficient. For a 3d composite with conductive filler materials distributed in an insulating matrix (such as the graphene-PVP composite), the equation can be rewritten in terms of volume fractions:


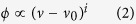


where 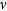 and 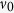 are the volume fraction and the critical volume fraction of the conducting objects, respectively^70^. Since this relation does not take into account of particle size, shape, orientation and their distribution uniformity inside a composite matrix, 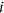 and 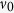 are empirically derived for a given system^70^. Modelling the graphene flakes as thin, circular, 2d conductive platelets and considering their 3d random distribution within the composite, it can be shown that^70^:


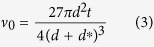


where 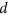 and 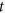 are the diameter and thickness of the platelets, respectively, and 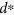 is the interplatelet distance. If 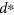 is shorter than the electron hopping distance through the non-conducting matrix, electron hopping takes place, facilitating formation of conductive pathways. The limit for single-step electron hopping is ~10 nm 71. Multistep electron hopping may take place for >10 nm. A critical value of ~1 *μ*m has been reported for some polymers with conductive fillers^71^,^72^. With ~4.5 nm average flake thickness and ~204 nm average lateral dimension estimated from AFM measurements and considering d^*^ ~ 10 nm-1 *μ*m, we get *v*_0_ ~ 0.486–0.003. This requires 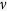 > 0.486 to ensure a conductive graphene-PVP composite. Bulk density of graphite is ~2.3 gcm^−3^. However, exfoliated graphene flakes can have a significantly lower density. Commercial graphene samples are typically quoted to have a density ranging from 0.03–0.4 gcm^−3^ 73. Assuming 0.4 gcm^−3^ for our UAPLE graphene, a >0.047 gL^−1^ concentration of graphene ink is required to form a conductive graphene-PVP composite. We stress that this estimation is dependent on further experimental determination of 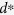 of PVP and the density of UALPE graphene. The graphene-PVP composite (*v* ~ 0.889) formed from this graphene ink (*c* ~ 0.40 gL^−1^) is therefore predicted to be conductive. This is experimentally confirmed with resistance measurement between the IDEs at room temperature, 40 °C and 70 °C, as shown in Fig. 6(a). The linear relationship confirms good Ohmic contact between the graphene-PVP composite and the gold IDEs. A control experiment is conducted by inkjet printing pure PVP solution, which shows negligible electrical conductivity, as expected.

The sensors are next tested using different relative humidity (*RH*) levels inside a stainless steel chamber. Initially the sensors are kept in dry air before they are exposed to eight different humidity levels (10–80% 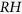). In typical experiments, the sensors are exposed to humid air for 20 minutes, followed by dry air purging for 30 minutes. Note that our set-up allows a maximum of 80% 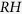 above which condensation of water vapor inside the test chamber starts to appear. The sensor response is defined as:


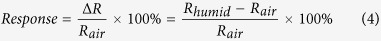


where 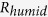 and 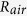 are the measured resistances exposed to humid conditions and dry air, respectively. Typical response of the sensor with the inkjet-printed graphene-PVP composite sensing layer when exposed to different 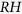 levels at room temperature is plotted in Fig. 6(b). We see an increased response with an increased 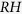. The sensitivity (defined as: Response/*RH*) achieved from the sensor varies from 0.3%/%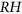 to 0.21%/%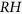 as 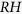 increases from 10% to 80%. We also observe a drift in the response baseline in the lower humidity levels (10–20%). This could be due to trapped water molecules within the sensing layer during the recovery from pre-exposures^74^.

To investigate the effect of temperature on the response, we have tested the sensors at three temperatures (room temperature, 40 °C and 70 °C). The temperature rise is achieved by using the on-chip microheater. The result is plotted in Fig. 6(c). We observe a reduced response with increasing temperature. We note that the baseline drift shown in Fig. 6(b) reduces with increase in temperature as the trapped water molecules desorb from the sensing layer faster. We further add that 40 °C and 70 °C are sufficiently low not to induce any discernible thermal stress on to the CMOS 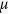
HP or the graphene-PVP film and affect the measurements.

To further investigate the influence of graphene volume fraction on the sensing layer, a series of 5 inks with 0.40, 0.32, 0.24, 0.16 and 0.08 gL^–1^ graphene concentration is prepared while keeping the PVP concentration constant. This gives the volume fraction 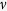 as 0.889, 0.865, 0.828, 0.762 and 0.615, respectively. With this *v* > 0.486, the deposited graphene-PVP composites after evaporation of IPA are predicated to be conductive. Sensors with these inks are then exposed to 40–60% 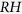. We observe an increased response with the increase of graphene concentration under all the 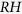 conditions; Fig. 6(d). We suggest that the graphene-PVP composite with more percolating pathways shows a larger change in resistance, leading to a higher response. The larger change may be subject to that more percolating pathways are broken due to the polymer swelling effect. However, this is yet to be conclusive, requiring further experimental work to understand the electrical conduction and percolation behavior of our graphene-PVP composite sensing layer.

**Figure 7:**
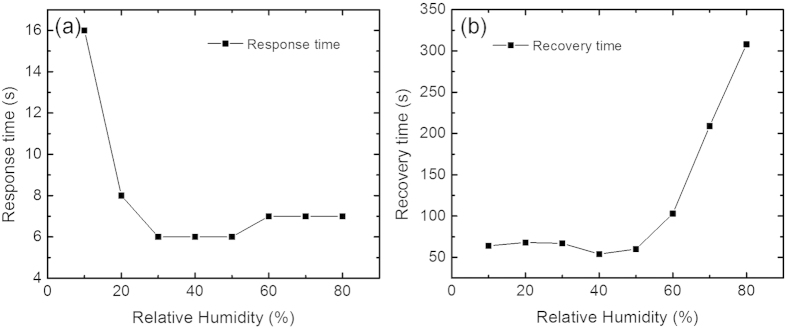
(**a**) Response time and (**b**) Recovery time at different *RH* levels at room temperature.

The sensor with 0.40 gL^−1^ graphene ink exhibits the highest response and is used to further investigate typical response and recovery times (defined as the time needed to reach 63% of the maximum response and to recover to the baseline, respectively). The response and recovery times measured at room temperature are reasonably fast, varying from ~6–16 s and ~60–300 s, respectively; Fig. 7(a,b). The slower recovery time at higher 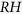 could be attributed to the presence of higher partial pressure of water vapour close to the surface of the sensing layer when the sensor recovers through the desorption of water molecules. It should be noted that long (humidity) 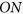 and 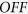 times were used to ensure that the device response reaches its saturated limit without any noticeable drift.

**Figure 8:**
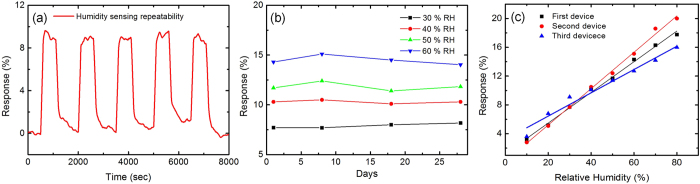
Sensing reproducibility of (**a**) one sensor during 5 sensing cycles (at 50% *RH*), (**b**) long-term stability, and (**c**) sensing variation of three different identical sensors.

Reproducibility of a sensor at different sensing cycles and of sensing over a long period are also investigated. We measure response of different sensors with the same design to investigate performance variations between the devices. All the sensors use 0.40 gL^−1^ ink and are tested at room temperature. The reproducibility of response is demonstrated at 50% 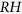 for five cycles; Fig. 8(a). The long-term stability of the sensor is investigated over a 4-week period. We do not observe a significant variation (~4%) of response at 30, 40, 50 and 60% 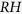 during this period; Fig. 8(b). The performance variation is investigated by measuring the response for three separate but identical sensors when exposed to different 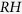 conditions, with a maximum variation of ~13%; Fig. 8(c). Figure 8(c) also shows a linear response of the sensors. We believe the variation could be further improved by optimizing the sensing layer and graphene flake dimensions for the development of reliable, reproducible, low power and compact sensors.

## Discussion

The adsorbed water molecules on graphene surface may disassociate to H^+^ and OH^−^ ions at the edges of graphene, similar to what is observed for graphene oxide samples^6^. These H^+^ ions may tunnel from one water molecule to another through hydrogen bonding, reducing the overall electrical resistance of the sensing layer^6^. On the other hand, the adsorbed water molecules on graphene surface act as electron donors^5^. As the electron density increases, the normally 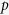-type graphene becomes more resistive. This p-type semiconductor nature of carbon materials is in agreement with reported results^75^,^76^. Meanwhile, PVP is a hygroscopic, electrically insulating polymer, absorbing up to 25% moisture at 75% 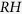, swelling the polymer^53^. We propose that moisture absorption and subsequently polymer swelling increases the distance between the graphene flakes, leading to a reduction of electrically percolating pathways through single hopping. We note this change in polymer volume and corresponding increase in distance between the adjacent graphene flakes may also depend on other factors such as chemical composition and graft density of the polymer film^77^. Among the two opposing effects on resistance, we suggest that effect of the 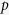-type semiconductor nature of graphene and the swelling and consequent reduction of percolating pathways dominate. This results in increased electrical resistance of the graphene-PVP composite sensing layer, enabling humidity sensing through the IDEs.

In summary, we have integrated functional graphene inks with CMOS MEMS technology to fabricate a resistive humidity sensor. Our formulation of the ink using a blend of hygroscopic, electrically insulating polymer with chemically pristine, conducting graphene nanoflakes offers an ideal sensing layer for use with an economic, compact and low power CMOS sensing platform. Through 3d percolation theory, we show that our ink formulation produces a conductive sensing layer. Upon humidity exposure, reduction in percolating networks in the sensing layer leads to an increased resistivity for humidity sensing. The response of the sensors are reproducible, with a maximum of ~13% variation among sensors at different 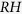 levels with stable performance (<4%) over a period of few weeks. The combination of CMOS and inkjet printing platform, with graphene and potentially, other nanomaterial based functional inks, opens exciting opportunities for mass produced CMOS based sensor systems for a tremendous variety of highly commercially valuable applications.

## Additional Information

**How to cite this article**: Santra, S. *et al*. CMOS integration of inkjet-printed graphene for humidity sensing. *Sci. Rep*. **5**, 17374; doi: 10.1038/srep17374 (2015).
